# Transcriptome Analysis of the Silkworm (*Bombyx mori*) by High-Throughput RNA Sequencing

**DOI:** 10.1371/journal.pone.0043713

**Published:** 2012-08-23

**Authors:** Yinü Li, Guozeng Wang, Jian Tian, Huifen Liu, Huipeng Yang, Yongzhu Yi, Jinhui Wang, Xiaofeng Shi, Feng Jiang, Bin Yao, Zhifang Zhang

**Affiliations:** 1 Biotechnology Research Institute, Chinese Academy of Agricultural Sciences, Beijing, China; 2 Feed Research Institute, Chinese Academy of Agricultural Sciences, Beijing, China; University of North Carolina at Charlotte, United States of America

## Abstract

The domestic silkworm, *Bombyx mori*, is a model insect with important economic value for silk production that also acts as a bioreactor for biomaterial production. The functional complexity of the silkworm transcriptome has not yet been fully elucidated, although genomic sequencing and other tools have been widely used in its study. We explored the transcriptome of silkworm at different developmental stages using high-throughput paired-end RNA sequencing. A total of about 3.3 gigabases (Gb) of sequence was obtained, representing about a 7-fold coverage of the *B. mori* genome. From the reads that were mapped to the genome sequence; 23,461 transcripts were obtained, 5,428 of them were novel. Of the 14,623 predicted protein-coding genes in the silkworm genome database, 11,884 of them were found to be expressed in the silkworm transcriptome, giving a coverage of 81.3%. A total of 13,195 new exons were detected, of which, 5,911 were found in the annotated genes in the Silkworm Genome Database (SilkDB). An analysis of alternative splicing in the transcriptome revealed that 3,247 genes had undergone alternative splicing. To help with the data analysis, a transcriptome database that integrates our transcriptome data with the silkworm genome data was constructed and is publicly available at http://124.17.27.136/gbrowse2/. To our knowledge, this is the first study to elucidate the silkworm transcriptome using high-throughput RNA sequencing technology. Our data indicate that the transcriptome of silkworm is much more complex than previously anticipated. This work provides tools and resources for the identification of new functional elements and paves the way for future functional genomics studies.

## Introduction

The domestic silkworm, *Bombyx mori*, has been intensively studied for the past several decades for its economic and academic value [1]. The most important economic value of the silkworm is in the production of silk. In 2010, the production value reached about 30.6 billion dollars in China’s silk industry [2]. Moreover, silkworms are used widely as bioreactors for the production of vaccines [3], enzymes [4], proteinaceous drugs [5], and other biomaterials. Silkworm has also been used as a model insect in the Lepidoptera order for biochemical, molecular genomic, and genetic research, and for pest control, particularly after its complete genome sequence was finished [6], [7]. A variety of methods, such as expressed sequence tags (ESTs) [8], [9], serial analysis of gene expression (SAGE) [10], [11], and microarrays [11], [12], were used to identify and determine the activity of the functional elements in the silkworm genome. A number of silkworm genomic resources are available; for example, the Silkworm Genome Database (SilkDB) [13] and KAIKObase, an integrated silkworm genome database and data mining tool [14]. However, because of limitations of the conventional technical approaches, the functional complexity of the silkworm transcriptome has not yet been fully elucidated.

The transcriptome is a complete set of RNA transcripts produced by the genome at any one time, and an understanding of the transcriptome is essential for interpreting the functional elements of the genome [15]. RNA-sequencing (RNA-Seq), which is based on the deep sequencing technology, is a powerful and cost-efficient tool for transcriptome analysis. Compared with other approaches for transcriptome analysis, RNA-Seq has the advantages of high throughput, high resolution, and low background noise [15], [16]. Moreover, the application of RNA-Seq technology to eukaryotic transcriptomes for transcript profiling has revealed an increasing number of novel transcripts and sequence variations as a result of alternative splicing (AS) [17], [18], [19], and gene fusion [19], [20]. Together, these results have indicated that the eukaryotic transcriptomes are more complex than previously believed.

To better understand the complexity of the silkworm transcriptome, RNA-Seq technology was applied to polyadenylated-enriched mRNAs from different stages and organs or tissues of the silkworm. Analysis of the results identified a substantial number of new exons and novel transcripts, which significantly improved the current genome annotation of silkworm. In addition, we analyzed the level of AS in the silkworm transcriptome and found that a far greater number of genes undergo AS than was previously identified. The transcriptome data also allowed accurate predictions of gene structures. Our results provide a global view of the silkworm transcriptome and pave the way for its further analysis.

## Results

### Summary of RNA-Seq Data Sets

To obtain a global view of the silkworm transcriptome and gene activity at single-nucleotide resolution, we performed high-throughput RNA-Seq experiments using Illumina sequencing technology on poly (A)–enriched RNAs from a pool sample that covered the representative developmental stages of silkworm eggs, larva, pupa, and moth (organs can be dissected at these stages). After removing the low-quality reads, a total of 33,025,188 paired-end reads with an average length of 100 bp (Figure S1) were obtained. The total length of the reads was about 3.3 gigabases (Gb), representing about a 7-fold coverage of the *B. mori* genome and a more than 100-fold coverage of the annotated transcriptome. All the short reads were mapped onto the *B. mori* genome using TopHat software [21]. We found that approximately 76% of the reads could be uniquely aligned to the genome; 45.45% of the reads mapped to known exons and 29.12% were located in predicted intergenic or intronic regions (Table S1).

### Overview of the Silkworm Transcriptome

**Figure 1:**
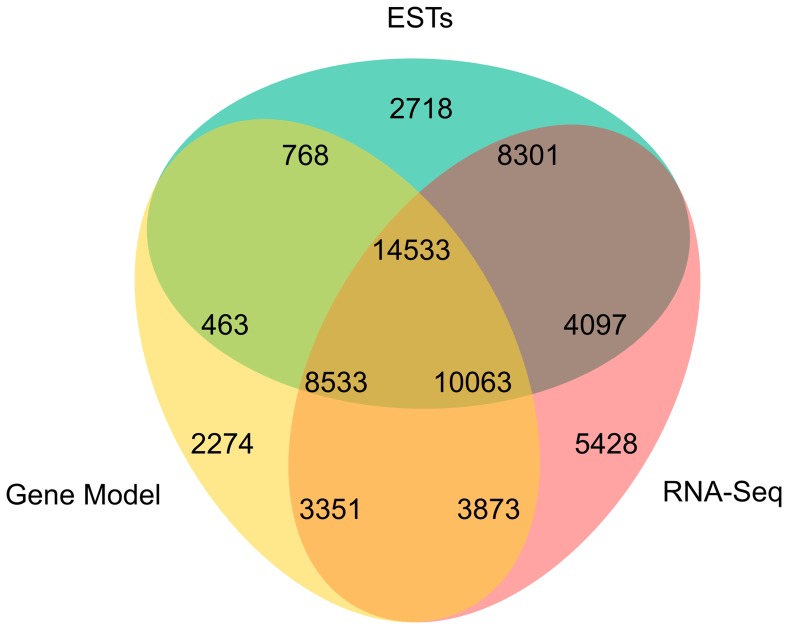
Comparison of transcripts detected using RNA-Seq with the publicly available ESTs. Numbers represent the sizes of the transcript sets. The EST sequence data were obtained from GenBank’s ESTdb; the RNA-Seq data are from the present study; the Gene Model data is from SilkDB.

A total of 23,461 transcripts were obtained from the reads that mapped to the genome sequence. Similarly, we mapped the silkworm EST sequences from GenBank’s ESTdb onto the *B. mori* genome, and found that more than 86.8% of the transcript regions identified from the EST alignments were present in our transcriptome data but more than 39.6% of the transcripts defined using the RNA-Seq reads were not detected by the EST mapping (Figure 1). Of the predicted 14,623 protein-coding silkworm genes that were built by merging different gene datasets using GLEAN in the SilkDB [13], 11,884 of them were found to be expressed in our data (Figure 1), which covered 81.3% of all the predicted genes.

**Figure 2:**
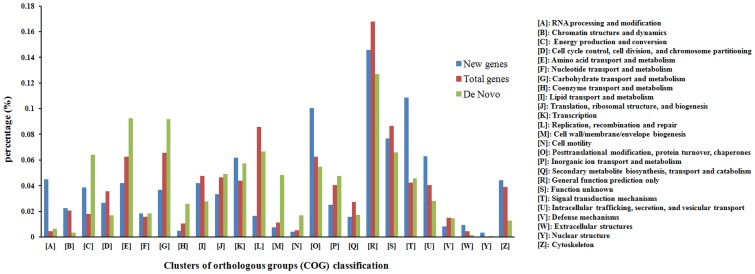
Clusters of orthologous groups (COG) classification of the silkworm genes. Total genes represent the combined of genes already annotated in the SilkDB and the new genes discovered in the present study; Novel genes represent the new genes identified by RNA-Seq in this study; De Novo represents the genes identified from the reads that could not mapped to the silkworm genome.

The identified transcripts were searched against the non-redundant protein database at the National Center for Biotechnology Information (NCBI) using BLASTX. We found a total of 16,740 sequences that had significant matches to known protein sequences. Classification of the unigenes into Cluster of Orthologous Groups of proteins (COG) categories showed that the “general function prediction” cluster was the most represented (19.6% of all the unigenes), followed by “replication, recombination and repair” (12.2% of all the unigenes). In addition, the “transcription”, “post-translational modification, protein turnover, chaperones” and “amino acid transport and metabolism” clusters were represented in a large portion of the unigenes (Figure 2).

Approximately 26.48% of reads fell within the intergenic regions of the annotated genes in the SilkDB. These reads may be derived from as yet unrecognized transcripts and/or noncoding RNAs. Read mapping and clustering revealed that 9,525 transcripts were identified, with an average length of 230 bp and an average depth of 92 (Table S2). Among them, 5,428 transcripts were found that did not match any of the annotated genes in the SilkDB or any of the EST sequences; these transcripts were defined as novel transcripts. After a homology search in NCBI’s non-redundant protein database using BLASTX, a total of 1,703 unique sequences with significant hits to known protein sequences were identified. A COG analysis showed that the cluster “general function prediction” was again the most represented (14.6% of all the annotated novel genes), but the gene functions distribution of the other COG clusters were different compared with the distribution for the total genes (Figure 2). The clusters “RNA processing and modification”, “function unknown”, and “extracellular structures” for the novel transcripts were much more highly represented compared with their representation in the COG clusters for the total genes (Figure 2).

**Figure 3:**
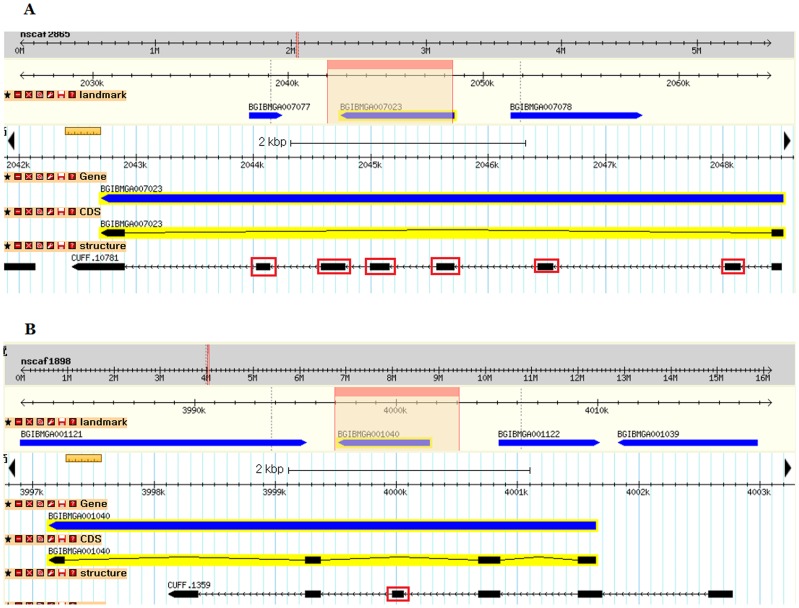
Examples of new exons detected in the silkworm transcriptome displayed using GBrowse in the SilkTransDB database. (A) Multiple new exons (red boxes) previously unidentified in the annotated intron of BGIBMGA007023 from SilkDB. (B) A single new exon (red box) previously unidentified in the annotated intron of BGIBMGA001040 from SilkDB.

**Figure 4:**
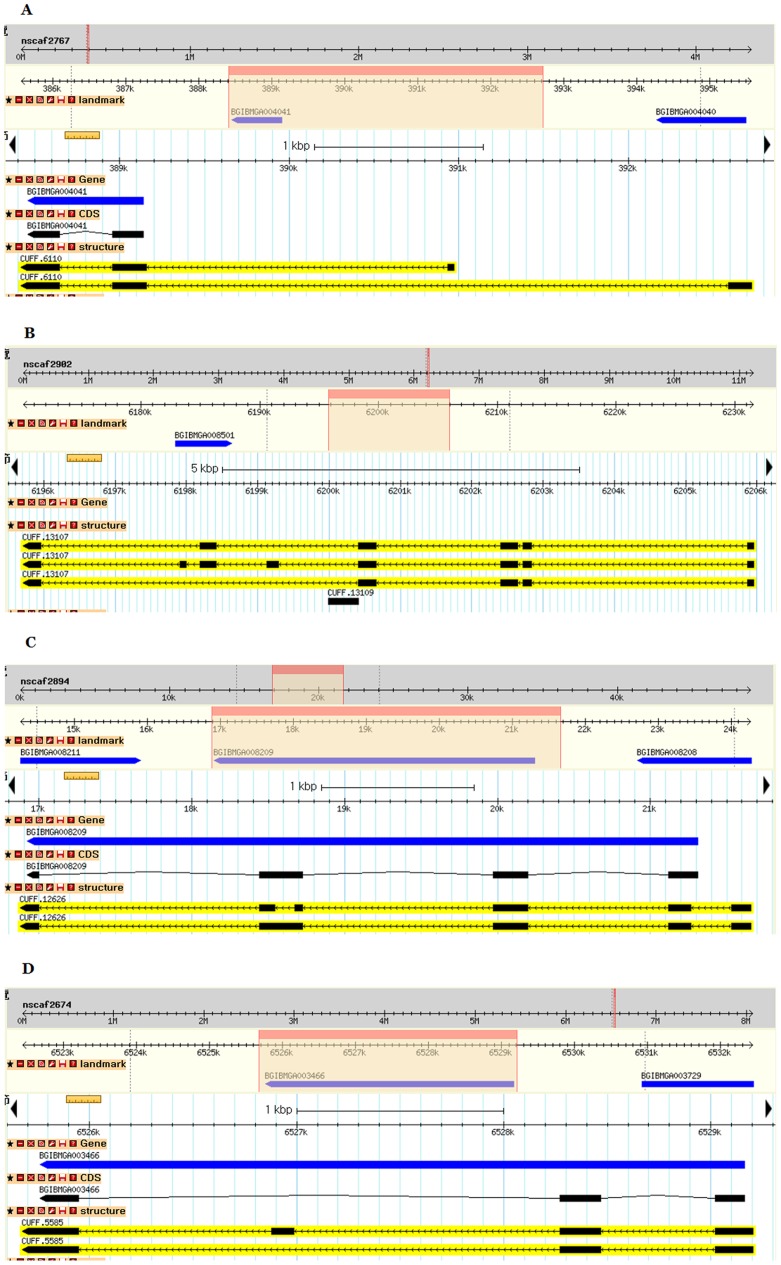
Examples of alternative splicing detected in the silkworm transcriptome displayed using GBrowse in the SilkTransDB database. (A) Alternative first exon of the CUFF.6110. (B) Multiple skipped exons of CUFF.13107. (C) Retained intron of the CUFF.12626. (D) Single skipped exon of CUFF.5585.

A total of 62,084 splice sites were identified in the transcript; the vast majority of them (97.6%) were Class I type (GT-AG/CT-AC) splice sites. A total of 13,195 new exons were detected, and 5,911 of them were identified in the annotated genes in the SilkDB (Table S3), suggesting that these genes may contain exons that were not recognized in the silkworm genome annotation process. For example, as illustrated in Figure 3, six new candidate exons were detected in the annotated gene BGIBMGA007023 (Figure 3A) and one was detected in BGIBMGA001040 (Figure 3B) based on the transcripts generated by transcriptome sequencing. These candidate exons were confirmed by RT–PCR (Figure S2). An analysis of AS in the transcriptome revealed that 3,247 genes could undergo alternative splicing. Four genes that were predicted to undergo AS events were selected for experimental validation (Figure 4). The AS events predicted in the four genes include alternative first exons (Figure 4A), skipped exons (Figure 4B, D), and retained introns (Figure 4C). The RT–PCR results suggested that these AS events exist (Figure S3).

The remaining 24.2% of the reads that could not be mapped to the silkworm genome were clustered and assembled into 37,408 contigs with an average length of 192.9 bp (Text S1). The largest contig was 4,300 bp long and 18,949 contigs were longer than 150 bp. BLASTX analysis showed that 11,199 contigs had hits to known proteins, a large number of which were silkworm proteins or proteins from species in the phylum of Arthropoda (Table S4). A COG analysis showed that the cluster “general function prediction” was again the most represented (12.68% of all the annotated genes), but the gene functions distribution of the other COG clusters were different compared with the distribution for the total genes and new genes (Figure 2). The clusters “energy production and conversion”, “coenzyme transport and metabolism”, “cell wall/membrane/envelope biogenesis”, and “cell motility” for the novel transcripts were much more highly represented compared with their representation in the COG clusters for the total genes and new genes (Figure 2).

### Validating the Integrity of the Transcriptome Using RT-PCR

To validate the integrity of the silkworm transcriptome, eight protein families, namely Yellow, 30 kDa protein, DnaJ, frizzled, Methuselah, aminopeptidase N protein, transposase and cuticle, were selected for analysis. All the known proteins in three of the families, Yellow, 30 kDa protein and DnaJ, were found in our transcriptome database; in addition, several new genes that have not yet been reported or annotated were found to encode proteins that fall into the Yellow (one new gene), 30 kDa protein (five new genes) and DnaJ families (six new genes) (Table S5). A total of Fifteen new genes from the eight families (Table S5) were validated using reverse transcription polymerase chain reaction (RT-PCR) (Figure S4). These results suggested that our transcriptome data have a high coverage.

### Expression Profiles of Seven New Genes in Different Tissues and/or Developmental Stages by Quantitative RT-PCR

**Figure 5:**
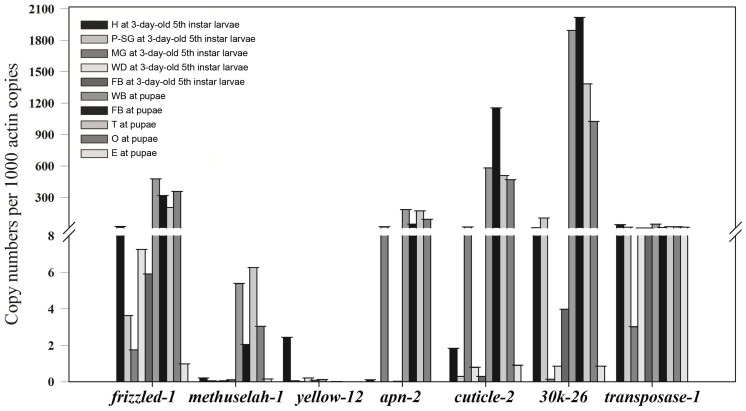
Expression profiles of seven genes in multiple tissues at different stages. The tissues were collected from third-day 5th-instar larvae and pupae. The copy number of all the genes multiplied by 1000 and then divided by the copy number of cytoplasmic actin (A3) mRNA was defined as the relative copy number. H (head), P-SG (posterior silk gland), MG (midgut), WD (wing discs), FB (fat body), (WB) wing bud, T (testis), O (ommateum) and E (egg).

The expression patterns of seven new genes from different protein families were selected for analysis by RT-PCR in head, posterior silk gland, midgut, wing disc, fat body at the 3-day-old 5th instar larvae stage; in wing bud, testis, ommateum, fat body at the pupae stage; and in egg at the adult stage (Figure 5). The mRNA copy numbers for each of the selected genes differed greatly in the selected tissues. For example, the *30K-26* mRNA copy numbers in the fat body at the stage of pupae were more than 506 and 14,076 times higher than its copy numbers in the fat body and midgut of 3-day-old 5th instar larvae, respectively. Moreover, the mRNA copy numbers for different genes in the same tissue were also different from each other. For example, in the fat body of pupae, mRNA copy numbers of *30K-26*, *Cuticle-2* and *frizzled1* were far higher than the copy numbers of the transposase-9225, apn3 and meth1 and no mRNA was found for *yellow12*.

### The Features and Functions of the Database

**Figure 6:**
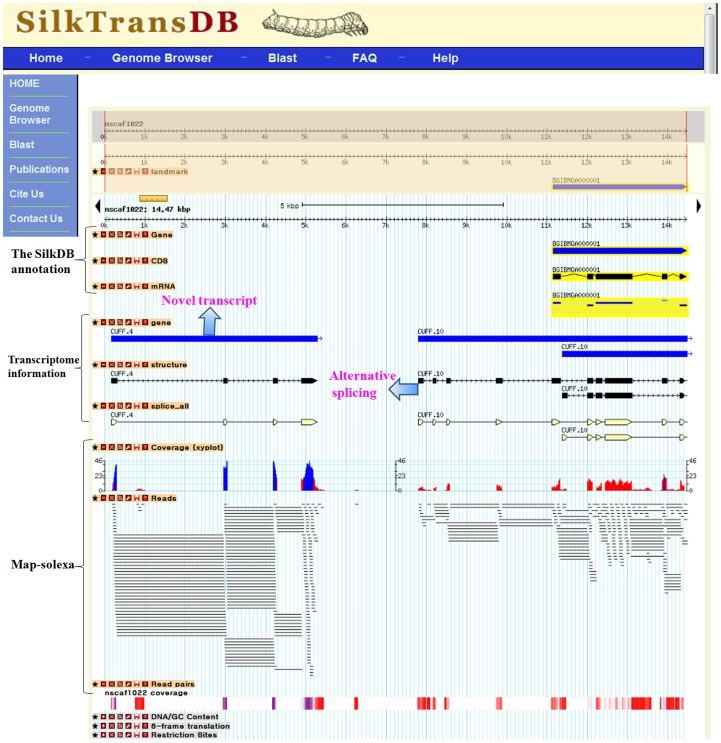
Screen shot of the interface of the SilkTransDB. The SilkDB annotation information, the mapped Solexa (map-solex) information, and the transcriptome information are identified by the vertical bar on the left of the screen. Users can use the interface to easily find the new annotation areas and other information, such as alternative splicing events, detected by the transcriptome analysis.

To promote data analysis, a website based database (SilkTransDB) was generated using GBrowse [22] to combine our transcriptome data with the annotated genomic information in the SilkDB. Users can use the SilkTransDB database server to perform BLAST searches or to browse the transcript information of individual genes by querying the transcription ID from our study or its gene ID in the SilkDB. SilkTransDB can display a gene-specific view and users can also obtain an overview of all the transcripts mapped to any user-selected position on a chromosome. Using the SilkTransDB interface, three kinds of information can be viewed: 1) SilkDB annotations that include CDS, gene, and mRNA information; 2) mapped Solexa data of coverage, reads, and read pairs; and 3) transcriptome information including gene, structure, and splice site (Figure 6). Using the combined information from SilkTransDB, users can easily identify genes that have not yet been annotated in the SilkDB. Users can also generate a view of the structure of an annotated gene based on the transcriptome information. This view may include the 3′ or/and 5′ ends of the gene that was not predicted by the SilkDB software. In addition, SilkTransDB can be used to identify new exons in a gene (Figure 3) or to predict an underlying AS event (Figure 4).

## Discussion

As a model insect, the genetics and molecular biology of silkworm has been well studied, especially after its genome was completed in 2004 [6], [7]. Transcriptome analysis can provide insights into functional genomics by its ability to interpret the functional elements of the genome, biological pathways, and molecular mechanisms. RNA-Seq is a recently developed technique that is powerful and cost efficient for the rapid identification and analysis of majority large part of the transcriptome [15], [16]. When this technique was applied to analyze the silkworm transcriptome, we found a substantial number of novel transcripts, far more than what had been found previously using conventional methods such as ESTs [8], [9]. In addition to novel transcripts, 5,911 new candidate exons were found in the annotated genes in the SilkDB. All these results suggested that our transcriptome data profoundly improved the existing gene annotations.

Alternative splicing is recognised as an important mechanism for proteomic diversity and functional complexity in higher eukaryotes [18], [23], [24]. Only a few AS events have been reported in silkworm. The genes that are known to be alternatively spliced include those that encode antitrypsin [25], arginases [26], lipophorin receptor [27], transformer-2 [28], chitinase [29], annexin IX [30], and GATA-type transcription factors [31]. By aligning ESTs with the silkworm genomic sequence, Xia *et al* [32] detected 277 AS forms in 235 silkworm genes. However, our analysis of the silkworm transcriptome data revealed that a greater number of genes may undergo AS than previously known. This finding suggests that AS may play an important role in the function regulation of silkworm genes.

SilkTransDB, generated using our transcriptome data combined with the annotated genomic information in SilkDB, helps users to easily find the improved annotation (for example, new transcripts, new exons, and ASs) that was obtained by the transcriptome analysis. As a novel and high-quality data resource, SilkTransDB is a valuable new tool for experimental biologists working on silkworm and related species. Because the sample we sequenced was pooled from more than 100 samples from different tissues at different stages of development, our database can provide an overview of the silkworm transcriptome but cannot be used to find differentially expressed genes or to determine the abundance the gene transcripts in different tissues or at developmental stages (Figure 5). This database will be further improved by including various annotated data on the silkworm genome in the future.

In conclusion, high-throughput paired-end RNA sequencing was used to explore the transcriptome of silkworm and a database was constructed to aid data analysis. A substantial number of novel transcripts, new exons, and AS events were found, which profoundly improved the existing gene annotations. With the combined information in the SilkTransDB, users can easily identify novel transcripts, obtain a view of the updated structure of an annotated gene, and find whether a gene may undergo an AS event or has newly identified exons. This work provides clues and resources for the identification of new genes and paves the way for future functional genomics studies.

## Materials and Methods

### Animals and RNA Extraction

The most popular commercial silkworm variety (Qiufeng X Baiyu) in China was raised and maintained in our laboratory. The insects were reared under normal conditions at 25°C at 70–80% relative humidity. Eggs (sampled every day at the incubation stage), ant worm, whole body of silkworm (except for middle gut contents) at the stage of the first to the fourth instar silkworm, middle-stage silkworm, and molting silkworm were used directly for total RNA extraction. Organs from at least 10 individual animal samples representing distinct stages of development were dissected out: blood, skin, fat body, tracheal hush, silk gland, Malpighian tubule, wing discs, foregut, midgut, hindgut, testis, ovarian, nervous system (brain, corpora cardiaca, corpora allata, subesophageal ganglion, prothorax ganglion, and abdominal ganglion), dorsal vessel, muscle of the fifth instar silkworm at the stages of newly molted silkworm, fifth instar 3d, and mature silkworm; blood, skin, fat body, tracheal hush, silk gland, malpighian tubule, wing discs, foregut, midgut, hindgut, brain, dorsal vessel, and muscle of silkworm prepupae; blood, skin, fat body, tracheal hush, silk gland, malpighian tubule, wing discs, foregut, midgut, hindgut, brain, dorsal vessel, muscle, female reproductive system, male reproductive system, and antennae at the first day and the compound eye darkening stages of the silkworm pupae; blood, skin, fat body, tracheal hush, malpighian tubule, midgut, brain, dorsal vessel, muscle, female reproductive system, male reproductive system, antennae, and compound eye of the newly eclosion moth. Total RNA was extracted from each sample using TRIzol Reagent or TRIzol LS Reagent for liquid samples (Invitrogen, Carlsbad, CA, USA) following the manufacturer’s protocol. To get high-quality RNAs from silkworm egg, the crude total RNA from egg samples were extracted using the one-step preparation method and then prepared using TRIzol following the manufacturer’s protocol. The integrity of the total RNA from each tissue was determined using gel electrophoresis. The total RNA quality was checked by measuring the absorbance at 260 and 280 nm (A260/280 of all samples >1.9).

### cDNA Library Preparation and Sequencing

Ten micrograms of purified total RNA from each sample (20 µg of the nervous system sample) were pooled together and used for library construction. Poly-A mRNAs were isolated from total RNA using Dynal magnetic beads (Invitrogen, Carlsbad, CA, USA), and then fragmented by heating at 94°C and used to synthesize double stranded cDNA with the random hexamer primer. The double stranded cDNA was adenylated at the 3′ end and ligated to the sequencing adapters. The ligated products were separated on 2% agarose gel; the 200–250 bp long fragments were selected and PCR amplified using Phusion polymerase (NEB, Ipswich, USA). Sequencing libraries were denatured with sodium hydroxide and diluted in hybridization buffer for loading onto one lane of an Illumina GA flow cell. Cluster formation, primer hybridization, and pair-end sequencing were performed using proprietary reagents according to the manufacturer’s recommended protocols.

### Sequence Analysis and Database Construction

The reference genome and annotation data of *B. mori* were downloaded from the SilkDB web site (http://www.silkdb.org/silkdb). The transcriptome/genome coverage was deduced using the transcriptome data in this study (3.3Gb) divided by the silkworm genome data (432 Mb) reported by The International Silkworm Genome Consortium [33]. To estimate expression levels and to discover novel genes and transcripts, the RNA-Seq reads generated on the Solexa platform were mapped to the silkworm genome using TopHat and determined using Cufflinks software [21]. To identify all potential splice sites, we searched for three types of splice of site (Class I: GT-AG/CT-AC; Class II: AT-AC/GT-AT; and Class III: CT-GC/GC-AG) in the intronic regions. To detect novel genes in the putative intergenic region, we compared the reference gene models and the reconstructed transcriptome from the RNA-Seq experiment. Transcript models determined by RNA-Seq without overlap were considered as novel transcripts. To assign annotations to the novel transcripts, we performed a similarity search against the Swiss-Prot and NCBI non-redundant protein sequence databases and the NCBI CCDS database with BLAST (E-value <1e^−5^). In addition, each unique sequence was annotated using COG. A set of in-house Perl scripts and EMBOSS (v6.4) were used for data manipulation. Reads that could not be matched to the silkworm genome were aligned and assembled into contigs. A website-based database (SilkTransDB) that integrated our RNA-Seq track signal data visualization with the annotation data in the SilkDB [13], was constructed using GBrowser [22].

### Validation of RNA-Seq by RT-PCR and Quantitative RT-PCR Analyses

Genes from various protein families, namely, the 30 kDa protein, Yellow, DnaJ, G protein-coupled receptor proteins (frizzled and Methuselah), aminopeptidase N protein, transposase and cuticle families, were selected to validate the newly discovered transcripts determined by RNA-Seq using RT-PCR. One microgram of the total RNA was reverse-transcribed into cDNA with a Reverse Transcription System kit (Promega, Madison, USA) according to the manufacturer’s protocol. Using the reverse transcription products as the templates, 30 cycles of amplification were performed for selected genes from three gene families (Table S5) using gene-specific primers (Table S6). In addition, primers were designed inside exons to detect different AS isoforms and new candidate exons (Table S6). The PCR products were separated on 1.5% agarose gel by electrophoresis.

Seven new genes from seven of the families, 30 kDa protein, Yellow, frizzled, Methuselah, aminopeptidase N protein, transposase and cuticle, were selected for analysis of their expression patterns in head, posterior silk glands, midgut, wing discs, fat body at 3-day-old 5th instar larvae; wing bud, testis, ommateum, fat body at pupae; egg at adult by quantitative RT-PCR. The RT-PCR reactions were carried out using the TOYOBO SYBR Green Real Time PCR master mix according to the manufacturer’s instructions. The experiments were repeated at least three times. The primers that were used for the RT-PCR are listed in Table S6. BmActin3 was used as an internal reference. The PCR products were separated using agarose gel-electrophoresis and purified using the Gel Extraction Kit (Qiagen, Weiden, Germany) according to the manufacturer’s instructions. The DNA concentrations were determined using a spectrophotometer (1 OD260 = 50 µg/ml). The mass of the DNA fragments was determined using the DNASTAR software package. RT-PCR was carried out using dilutions from 1011 copies/µl to 105 copies/µl to obtain a standard curve, which was then used to calculate the absolute copy numbers of the relevant genes. Comparison with the internal reference gave the relative copy numbers.

## Supporting Information

Figure S1 **Analysis of the sequence quality of the silkworm transcriptome.** **a.** Per base sequence content; **b.** Per base sequence quality; **c.** Base quality distribution; **d.** Per base high quality sequence (Q >20) content.
(PDF)

Figure S2 **RT-PCR experimental validation of the selected four genes with new exons. a. New exons in the annotated genes.** A: Multiple new exons in the BGIBMGA007023; B: Single new exon in the BGIBMGA001040; C: Multiple new exons in the BGIBMGA001090; D: Single new exon in the BGIBMGA010106. **b. RT-PCR validation of four genes.** M: DNA marker; 1: BGIBMGA007023; 2: BGIBMGA001040; 3: BGIBMGA001090; 4: BGIBMGA010106.
(PDF)

Figure S3 **RT-PCR experimental validation of the selected genes underlying alternative splicing events. a. Alternative splicing events of six genes.** A: multiple skipped exon of CUFF.13107; B: retained intron of the CUFF.12626; C: alternative first exon of the CUFF.6110; D: single skipped exon of CUFF.5585; E: alternative first exon of the CUFF.6350; F: alternative last exon of the CUFF.10775. **b. Alternative splicing validation of six genes.** M: DNA marker; 1: Three AS forms of CUFF.13107; 2: Two AS forms of CUFF.12626; 3 and 4: Two AS forms of CUFF.6110; 5: Two AS forms of CUFF.5585. 6 and 7: Two AS forms of CUFF.6350; 8 and 9: Two AS forms of CUFF.10775.
(PDF)

Figure S4 **RT-PCR experimental validation of the transcriptional activity of new genes in eight protein families.** M, DNA marker; 1, new gene Bm-Yellow-12 of Yellow protein family; 2, new gene Bm-Yellow-fa of Yellow protein family; 3, new gene Bm30 K-17 of 30 kD protein family; 4, new gene Bm30 K-26 of 30 kD protein family; 5, new gene DnaJ18 of Dna J protein family; 6, new gene DnaJ26 of Dna J protein family. 7, new gene Transposase-1 of Transposase family; 8, new gene Transposase-2 of Transposase family; 9, new gene Cuticle-2 of Cuticle family; 10, new gene Cuticle-6 of Cuticle family; 11, new gene Frizzled-1 of Frizzled family; 12, new gene Frizzled-4 of Frizzled family; 13, new gene Methuselah -2 of Methuselah family; 14, new gene Methuselah -1 of Methuselah family; 15, new gene APN2 of aminopeptidase N protein family.
(PDF)

Table S1 **A summary of the transcriptome sequence data.**
(DOC)

Table S2 **Total length of the reads that mapped to the intergenic regions of the silkworm genome.**
(DOC)

Table S3 **Details of the 5,911 new exons found in the annotated silkworm genes.**
(XLS)

Table S4 **The BLASTX results of the contigs generated from the reads that could not be mapped to the silkworm genome.**
(XLS)

Table S5 **Lists of the genes from SilkDB annotated as belonging to three gene families that were selected for validating the integrity of the silkworm transcriptome and for new genes validation using RT–PCR.**
(DOC)

Table S6 **Primers used for new gene, alternative splicing and new exon validation.**
(DOC)

Text S1 **Lists of the 37,408 contigs assembled from reads could not be mapped to the silkworm genome.**
(TXT)
