# Supplementary material for: Transcriptome Analysis of the Silkworm (Bombyx mori) by High-Throughput RNA Sequencing
Source: PLoS One. 2012 Aug 23;7(8):e43713. doi: 10.1371/journal.pone.0043713 (PMC3426547; doi:10.1371/journal.pone.0043713)
Supplement: Figure S1 — Analysis of the sequence quality of the silkworm transcriptome. a. Per base sequence content; b. Per base sequence quality; c. Base quality distribution; d. Per base high quality sequence (Q >20) content. (PDF) [file pone.0043713.s001.pdf]

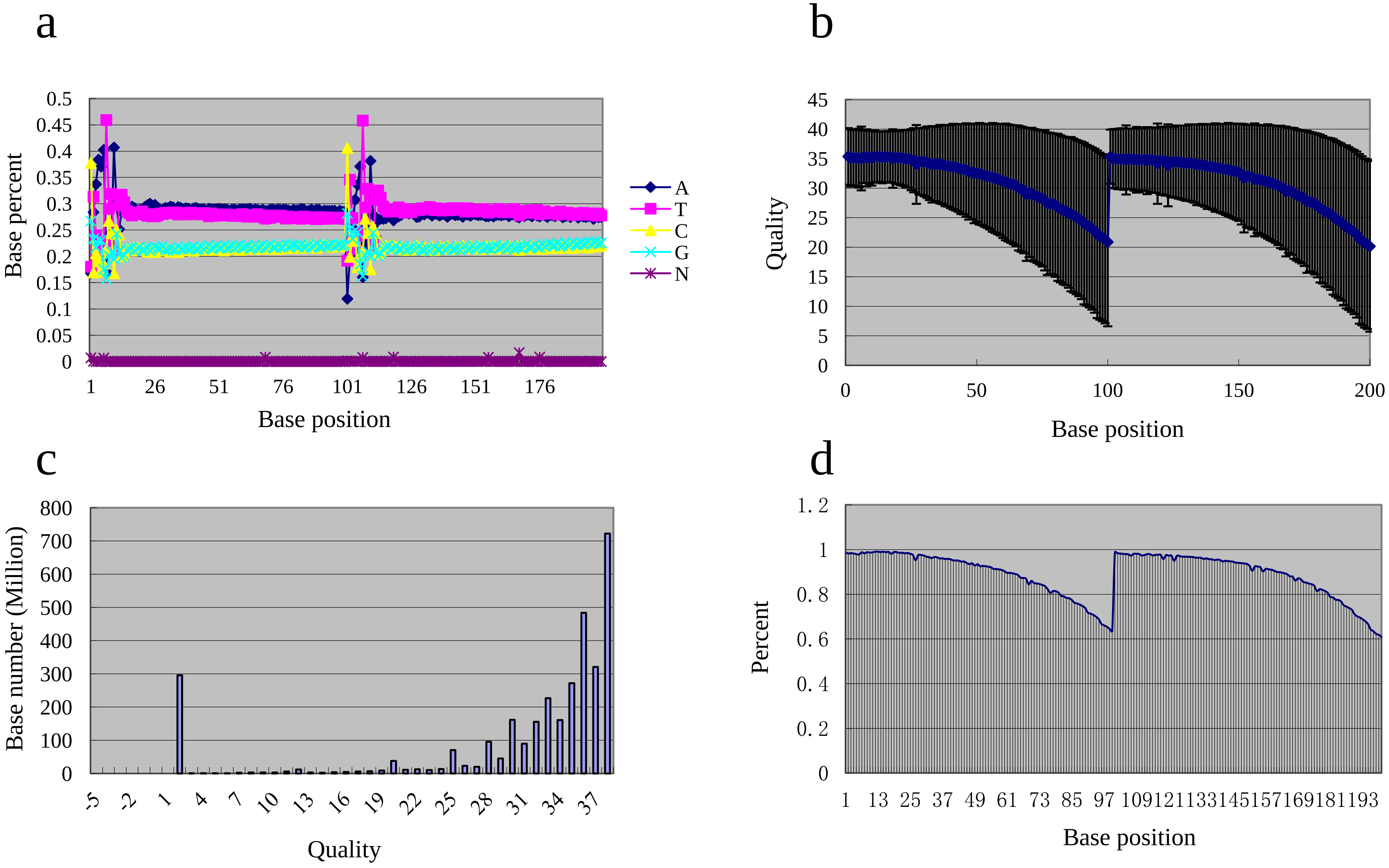

Figure S1.Sequence quality analysis of the silkworm transcriptome. a. per base sequence content; **b.** per base sequence quality; **c.** base quality distribution; **d.** per base high quality sequence (Q>20)content
