# Supplementary material for: Transcriptome Analysis of the Silkworm (Bombyx mori) by High-Throughput RNA Sequencing
Source: PLoS One. 2012 Aug 23;7(8):e43713. doi: 10.1371/journal.pone.0043713 (PMC3426547; doi:10.1371/journal.pone.0043713)
Supplement: Figure S2 — RT-PCR experimental validation of the selected four genes with new exons. a. New exons in the annotated genes. A: Multiple new exons in the BGIBMGA007023; B: Single new exon in the BGIBMGA001040; C: Multiple new exons in the BGIBMGA001090; D: Single new exon in the BGIBMGA010106. b. RT-PCR validation of four genes. M: DNA marker; 1: BGIBMGA007023; 2: BGIBMGA001040; 3: BGIBMGA001090; 4: BGIBMGA010106. (PDF) [file pone.0043713.s002.pdf]

a

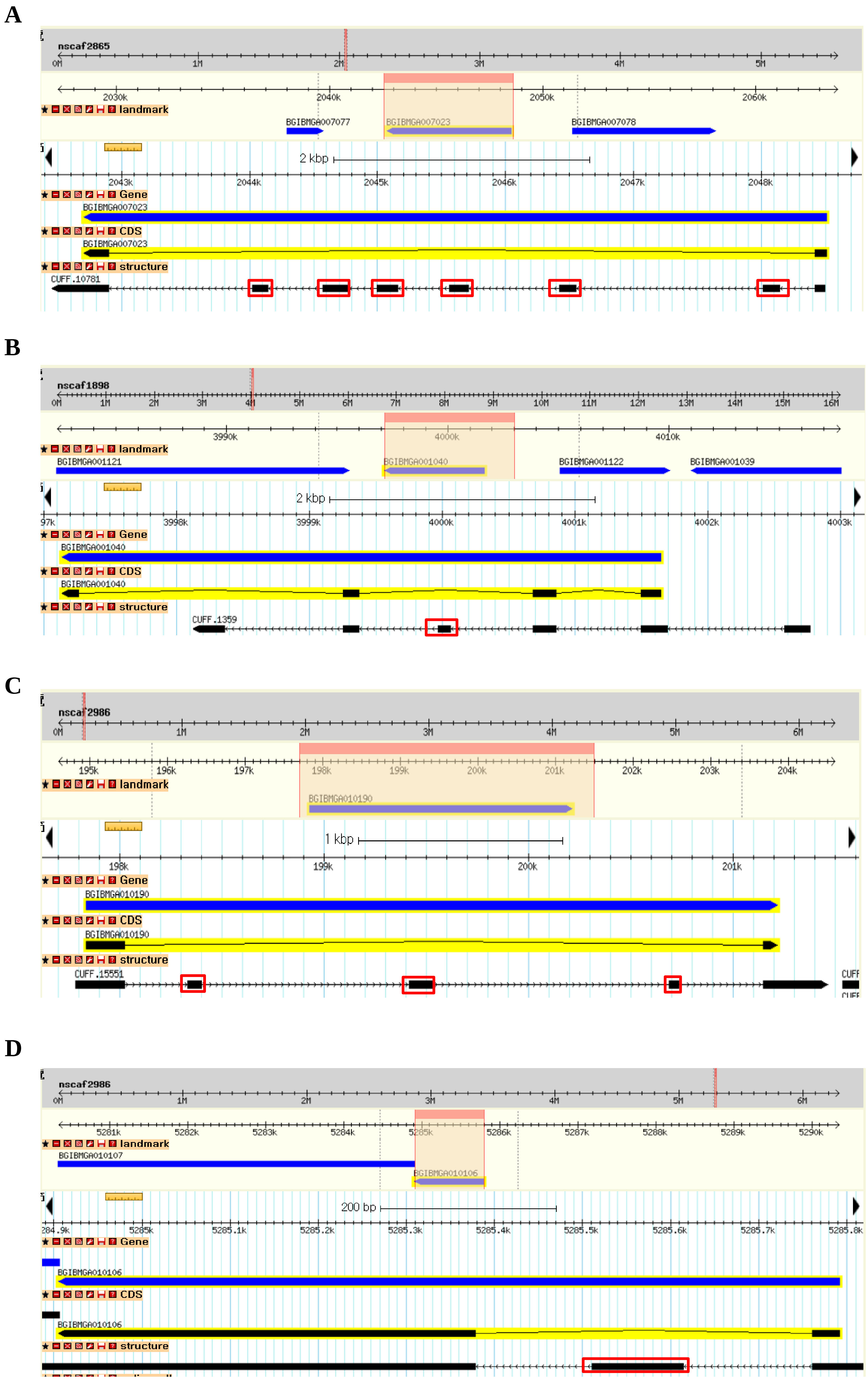

A
nscaf2865
landmark
BGIBMGA007077
BGIBMGA007023
BGIBMGA007078
2 kbp
Gene
BGIBMGA007023
CDS
BGIBMGA007023
structure
CUFF.10781
B
nscaf1898
landmark
BGIBMGA001121
BGIBMGA001040
BGIBMGA001122
BGIBMGA001039
2 kbp
Gene
BGIBMGA001040
CDS
BGIBMGA001040
structure
CUFF.1359
C
nscaf2986
landmark
BGIBMGA010190
1 kbp
Gene
BGIBMGA010190
CDS
BGIBMGA010190
structure
CUFF.15551
D
nscaf2986
landmark
BGIBMGA010107
BGIBMGA010106
200 bp
Gene
BGIBMGA010106
CDS
BGIBMGA010106
structure

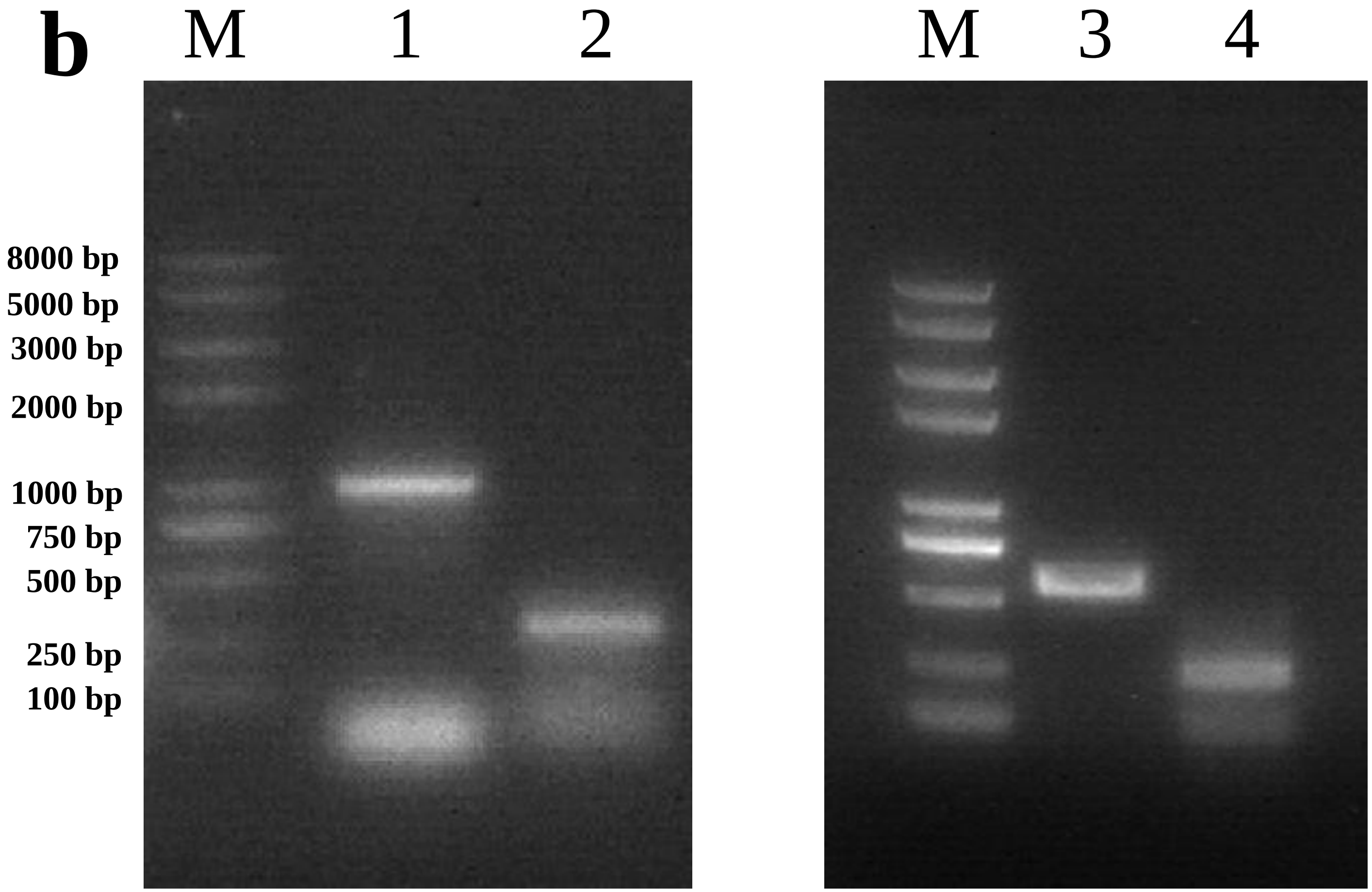

Figure S2. RT-PCR experimental validation of the selected four genes with new exons. a. New exons in the annotated genes. A: Multiple new exons in the BGIBMGA007023; B: Single new exon in the BGIBMGA001040; C: Multiple new exons in the BGIBMGA001090; D: Single new exon in the BGIBMGA010106. **b. RT-PCR validation of four genes.** M: DNA marker; 1: BGIBMGA007023; 2: BGIBMGA001040; 3: BGIBMGA001090; 4: BGIBMGA010106.
