# Supplementary material for: Transcriptome Analysis of the Silkworm (Bombyx mori) by High-Throughput RNA Sequencing
Source: PLoS One. 2012 Aug 23;7(8):e43713. doi: 10.1371/journal.pone.0043713 (PMC3426547; doi:10.1371/journal.pone.0043713)
Supplement: Figure S3 — RT-PCR experimental validation of the selected genes underlying alternative splicing events. a. Alternative splicing events of six genes. A: multiple skipped exon of CUFF.13107; B: retained intron of the CUFF.12626; C: alternative first exon of the CUFF.6110; D: single skipped exon of CUFF.5585; E: alternative first exon of the CUFF.6350; F: alternative last exon of the CUFF.10775. b. Alternative splicing validation of six genes. M: DNA marker; 1: Three AS forms of CUFF.13107; 2: Two AS forms of CUFF.12626; 3 and 4: Two AS forms of CUFF.6110; 5: Two AS forms of CUFF.5585. 6 and 7: Two AS forms of CUFF.6350; 8 and 9: Two AS forms of CUFF.10775. (PDF) [file pone.0043713.s003.pdf]

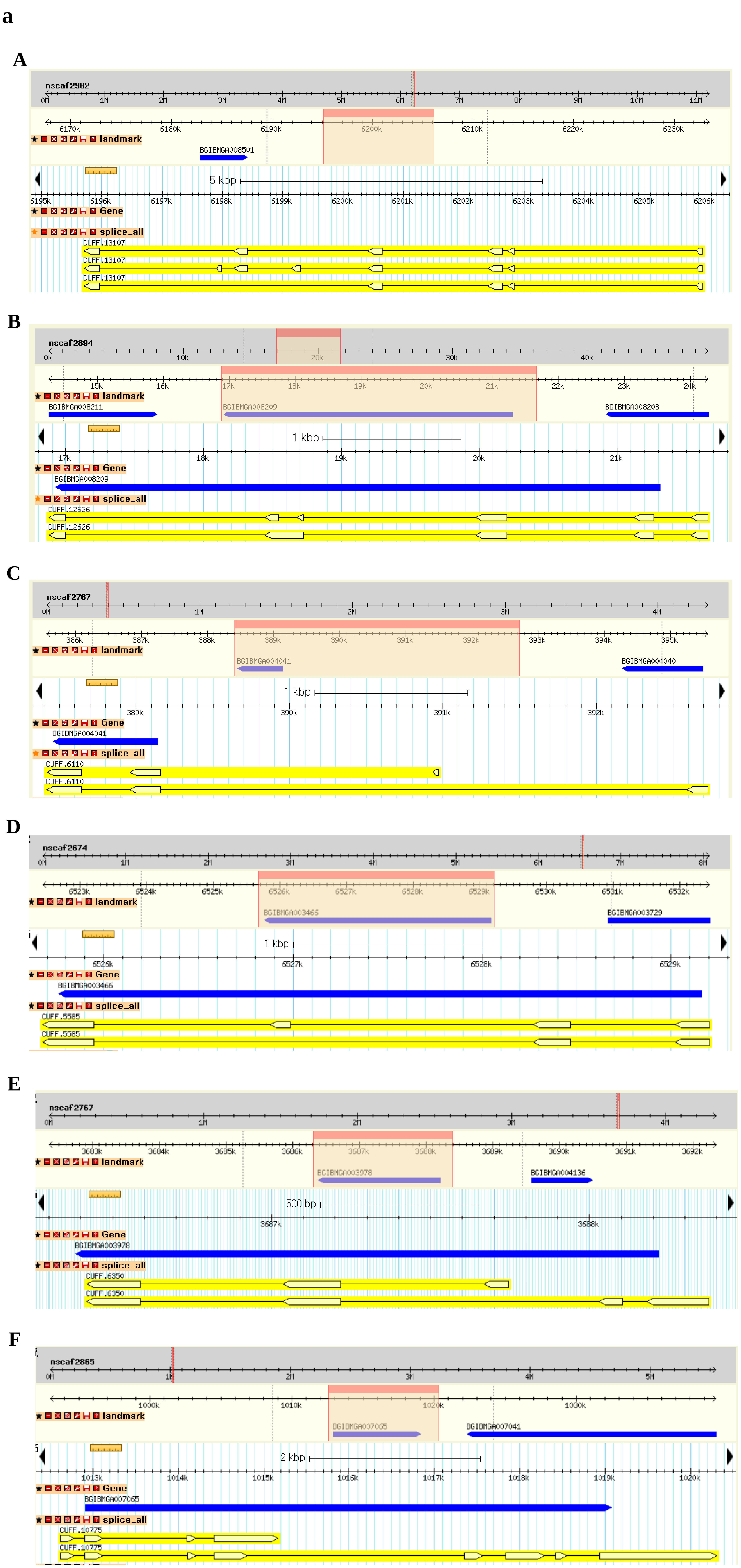
a
A
nscaf2902
landmark
BGIBMGA008501
5 kbp
Gene
splice_all
CUFF.13107
CUFF.13107
CUFF.13107
B
nscaf2894
landmark
BGIBMGA008211
BGIBMGA008209
BGIBMGA008208
1 kbp
Gene
BGIBMGA008209
splice_all
CUFF.12626
CUFF.12626
C
nscaf2767
landmark
BGIBMGA004041
BGIBMGA004040
1 kbp
Gene
BGIBMGA004041
splice_all
CUFF.6110
CUFF.6110
D
nscaf2674
landmark
BGIBMGA003466
BGIBMGA003729
1 kbp
Gene
BGIBMGA003466
splice_all
CUFF.5585
CUFF.5585
E
nscaf2767
landmark
BGIBMGA003978
BGIBMGA004136
500 bp
Gene
BGIBMGA003978
splice_all
CUFF.6350
CUFF.6350
F
nscaf2865
landmark
BGIBMGA007065
BGIBMGA007041
2 kbp
Gene
BGIBMGA007065
splice_all
CUFF.10775
CUFF.10775

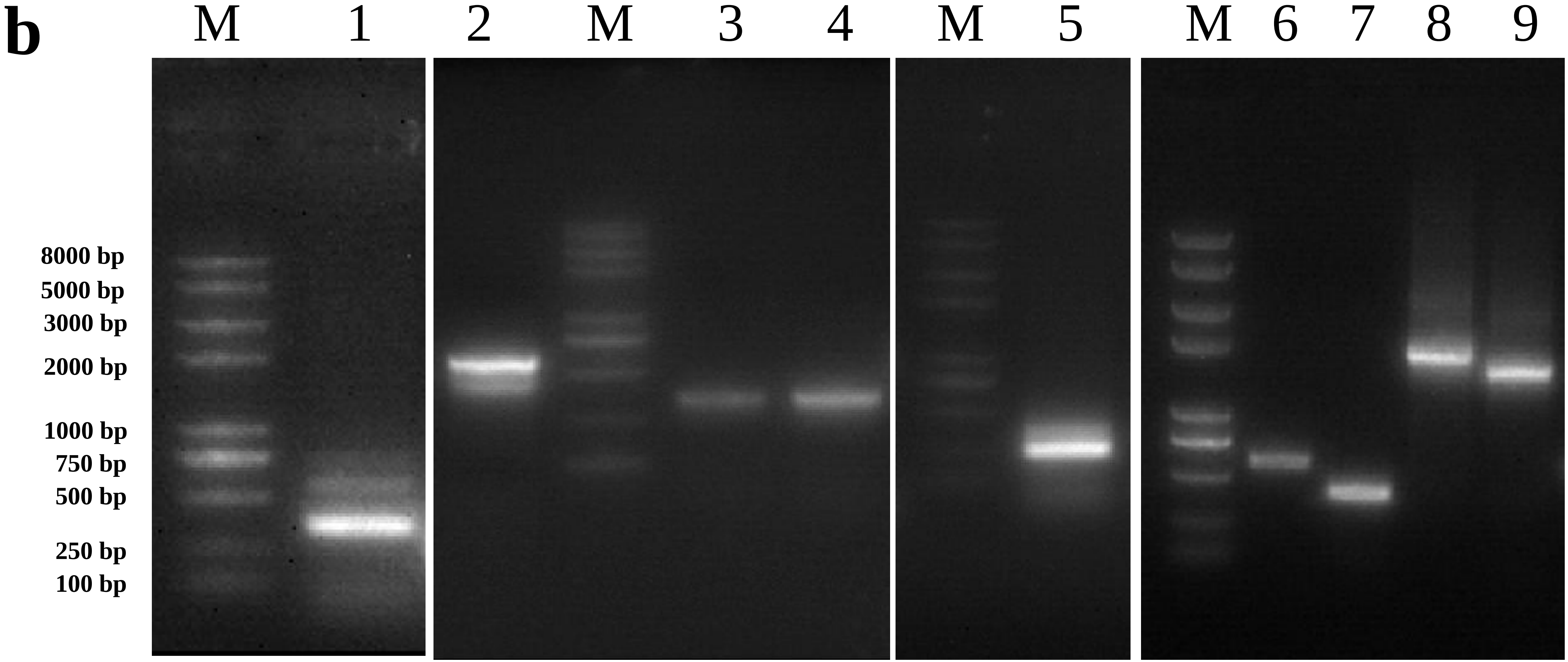

Figure S3. RT-PCR experimental validation of the selected genes underlying alternative splicing events. a. Alternative splicing events of six genes. A: multiple skipped exon of CUFF.13107; B: retained intron of the CUFF.12626; C: alternative first exon of the CUFF.6110; D: single skipped exon of CUFF.5585; E: alternative first exon of the CUFF.6350; F: alternative last exon of the CUFF.10775. **b. Alternative splicing validation of six genes.** M: DNA marker; 1: Three AS forms of CUFF.13107; 2: Two AS forms of CUFF.12626; 3 and 4: Two AS forms of CUFF.6110; 5: Two AS forms of CUFF.5585. 6 and 7: Two AS forms of CUFF.6350 ; 8 and 9: Two AS forms of CUFF.10775.
