# Supplementary material for: Transcriptome Analysis of the Silkworm (Bombyx mori) by High-Throughput RNA Sequencing
Source: PLoS One. 2012 Aug 23;7(8):e43713. doi: 10.1371/journal.pone.0043713 (PMC3426547; doi:10.1371/journal.pone.0043713)
Supplement: Figure S4 — RT-PCR experimental validation of the transcriptional activity of new genes in eight protein families. M, DNA marker; 1, new gene Bm-Yellow-12 of Yellow protein family; 2, new gene Bm-Yellow-fa of Yellow protein family; 3, new gene Bm30 K-17 of 30 kD protein family; 4, new gene Bm30 K-26 of 30 kD protein family; 5, new gene DnaJ18 of Dna J protein family; 6, new gene DnaJ26 of Dna J protein family. 7, new gene Transposase-1 of Transposase family; 8, new gene Transposase-2 of Transposase family; 9, new gene Cuticle-2 of Cuticle family; 10, new gene Cuticle-6 of Cuticle family; 11, new gene Frizzled-1 of Frizzled family; 12, new gene Frizzled-4 of Frizzled family; 13, new gene Methuselah -2 of Methuselah family; 14, new gene Methuselah -1 of Methuselah family; 15, new gene APN2 of aminopeptidase N protein family. (PDF) [file pone.0043713.s004.pdf]

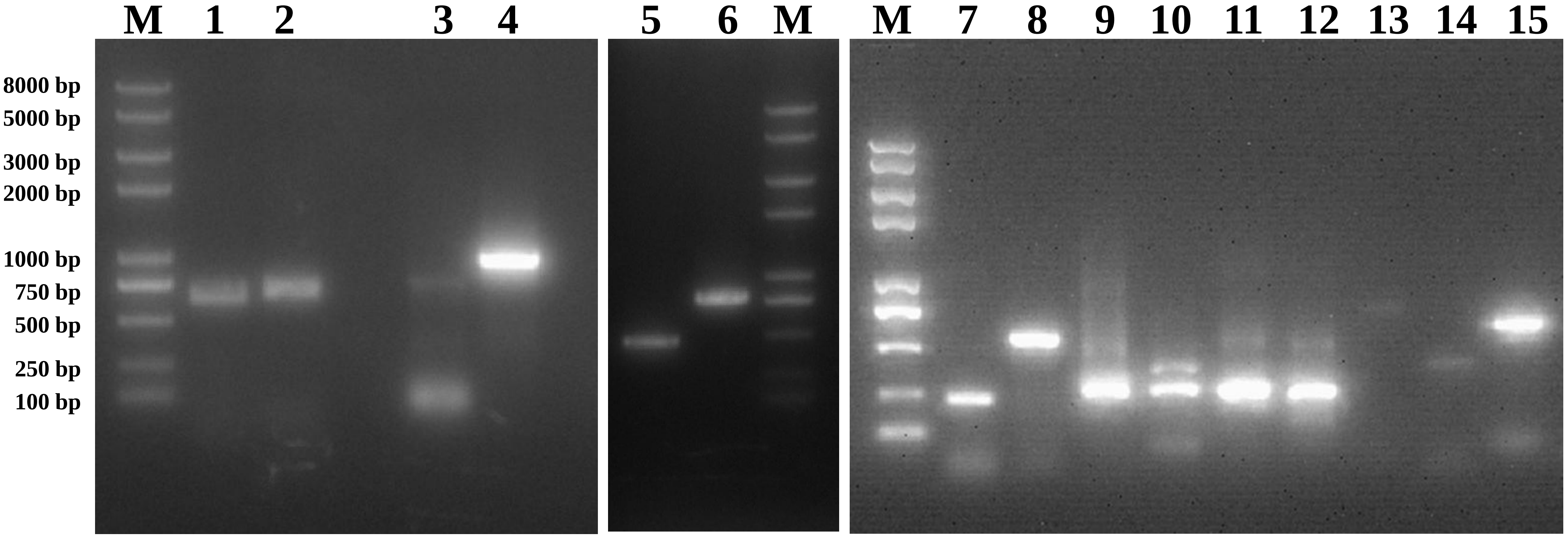

Figure S4. RT-PCR experimental validation of the transcriptional activity of new genes in the Yellow, 30kD protein, and Dna J protein families. M, DNA marker; 1, new gene Bm-Yellow-12 of Yellow protein family; 2, new gene Bm-Yellow-fa of Yellow protein family; 3, new gene Bm30K-17 of 30kD protein family; 4, new gene Bm30K-26 of 30kD protein family; 5, new gene DnaJ18 of Dna J protein family; 6, new gene DnaJ26 of Dna J protein family. 7, new gene Transposase-1 of Transposase family; 8, new gene Transposase-2 of Transposase family; 9, new gene Cuticle-2 of Cuticle family; 10, new gene Cuticle-6 of Cuticle family; 11, new gene Frizzled-1 of Frizzled family; 12, new gene Frizzled-4 of Frizzled family; 13, new gene Methuselah -2 of Methuselah family; 14, new gene Methuselah -1 of Methuselah family; 15, new gene APN2 of aminopeptidase N protein family.
