# Supplementary material for: Transcriptome Analysis of the Silkworm (Bombyx mori) by High-Throughput RNA Sequencing
Source: PLoS One. 2012 Aug 23;7(8):e43713. doi: 10.1371/journal.pone.0043713 (PMC3426547; doi:10.1371/journal.pone.0043713)
Supplement: Table S1 — A summary of the transcriptome sequence data. (DOC) [file pone.0043713.s005.doc]

**Table S1 A summary of the transcriptome sequence data**

|  | Data size | Percentage (%) of the total |
| --- | --- | --- |
| Total data size | 3.3 Gb | 100.00 |
| Mapped to genome regions | 2.5 Gb | 75.76 |
| Mapped to gene regions | 1.6 Gb | 48.48 |
| Mapped to exon regions | 1.5 Gb | 45.45 |
| Mapped to intron regions | 87 Mb | 2.64 |
| Mapped to intergenic regions | 874 Mb | 26.48 |
| Mapped to rRNA regions | 5.3 Mb | 0.16 |
| Mapped to tRNA regions | 4.7 Mb | 0.14 |
