# Supplementary material for: Transcriptome Analysis of the Silkworm (Bombyx mori) by High-Throughput RNA Sequencing
Source: PLoS One. 2012 Aug 23;7(8):e43713. doi: 10.1371/journal.pone.0043713 (PMC3426547; doi:10.1371/journal.pone.0043713)
Supplement: Table S2 — Total length of the reads that mapped to the intergenic regions of the silkworm genome. (DOC) [file pone.0043713.s006.doc]

**Table S2** Total length of the reads that mapped to the intergenic regions of the silkworm genome

| Mapped to intergenic regions: | 874 Mb |
| --- | --- |
| Coverage regions | 6 Mb |
| Total data size | 550 Mb |
| Average sequence length | 230 bp |
| The longest sequence length | 4877 bp |
| The shortest sequence length | 91 bp |
| The max coverage | 23502 |
| The mini coverage times | 20 |
| Average coverage times | 92 |
| Number of transcripts | 5428 |
