# Supplementary material for: Transcriptome Analysis of the Silkworm (Bombyx mori) by High-Throughput RNA Sequencing
Source: PLoS One. 2012 Aug 23;7(8):e43713. doi: 10.1371/journal.pone.0043713 (PMC3426547; doi:10.1371/journal.pone.0043713)
Supplement: Table S5 — Lists of the genes from SilkDB annotated as belonging to three gene families that were selected for validating the integrity of the silkworm transcriptome and for new genes validation using RT–PCR. (DOC) [file pone.0043713.s009.doc]

**Table S5-1** Yellow family of protein coding genes including the new potential genes (in bold) detected in our silkworm transcriptome.

| **Protein name** | **mRNA ID** | **Length (bp)** | **SilkDB ID^b^** | **Position on**  **the genome** | **Accession number** |
| --- | --- | --- | --- | --- | --- |
| Bm-Yellow-f | CUFF.22018 | 1323 | BGIBMGA014224 | nscaf98: 1519254-1523351 | JN977503 |
| Bm-Yellow-b | CUFF.560 | 1374 | BGIBMGA007072 | nscaf2865: 1675394-1677406 | JN977504 |
| Bm-Yellow-c | CUFF.10766 | 1224 | BGIBMGA000418 | nscaf1681: 2713396-2726396 | JN977505 |
| Bm-Yellow-4d | CUFF.11321 | 1341 | BGIBMGA007254 | nscaf2876: 1468499-1482863 | JN977506 |
| **Bm-Yellow-fa^1,2^** | **CUFF.2513** | **1038** | **——** | **nscaf2176: 1367119-1379950** | **JN977507** |
| Bm-Yellow-fd^2^ | CUFF.2506 | 1257 | **——** | nscaf2176: 1336459-1354092 | JN977508 |
| Bm-Yellow | CUFF.1474 | 1454 | BGIBMGA001149 | nscaf1898: 6287250-6296770 | JN977509 |
| Bm-Yellow-8 | CUFF.21532 | 1341 | BGIBMGA014026 | nscaf2759: 16236-542 | JN977510 |
| Bm-Yellow-f2 | CUFF.21539 | 1341 | BGIBMGA014032 | nscaf3106: 222927-242782 | JN977511 |
| Bm-Yellow-e | CUFF.11182 | 1221 | BGIBMGA007253 | nscaf2876: 1501998-1524720 | JN977512 |
| Bm-Yellow-11 | CUFF.21539 | 885 | BGIBMGA014032 | nscaf3106: 178715-186746 | JN977513 |
| **Bm-Yellow-12^1^** | **CUFF.11326** | **1530** | **BGIBMGA007255** | **nscaf2876: 1440921-1455749** | **JN977514** |
| Bm-Yellow-13 | CUFF.11322 | 1197 | BGIBMGA007256 | nscaf2876: 1427760-1430609 | JN977515 |
| Bm-Yellow-14^2^ | CUFF.21539 | 957 | **——** | nscaf3106: 211551-222937 | JN977516 |
| Bm-Yellow-15 | CUFF.5919 | 1359 | BGIBMGA003918 | nscaf2759: 84694-93763 | JN977517 |
| Bm-Yellow-16 | CUFF.21539 | 1341 | BGIBMGA014032 | nscaf3106: 207499-222967 | JN977518 |

^1^ New Yellow family genes that were detected in our silkworm transcriptome.

^2^New Yellow family genes found in the transcriptome that were not represented in the SilkDB

**Table S5-2** 30kD protein family of protein coding genes including the new potential genes (in bold) detected in our silkworm transcriptome.

| **Protein name** | **mRNA ID** | **Length (bp)** | **SilkDB ID** | **Position on**  **the genome** | | **Accession number** | |
| --- | --- | --- | --- | --- | --- | --- | --- |
| Bm30K-1 | CUFF.23774 | 1097 | BGIBMGA004396 | nscaf2795:  1655987-1657586 | JN977519 | |  |
| Bm30K-2 | CUFF.23788 | 2234 | BGIBMGA004397 | nscaf2795: 1643523-1647221 | JN977520 | |  |
| Bm30K-3 | CUFF.14597 | 1425 | BGIBMGA004400 | nscaf2795: 1619174-1620598 | JN977521 | |  |
| Bm30K-4 | CUFF.6741 | 908 | BGIBMGA004401 | nscaf2795: 1600934-1601841 | JN977522 | |  |
| Bm30K-5 | CUFF.6799 | 1279 | BGIBMGA004404 | nscaf2795: 1563844-1566685 | JN977523 | |  |
| Bm30K-6 | CUFF.8904 | 1283 | BGIBMGA004395 | nscaf2795: 1671934-1674134 | JN977524 | |  |
| Bm30K-7 | CUFF.9945 | 874 | BGIBMGA004399 | nscaf2795: 1629877-1631545 | JN977525 | |  |
| Bm30K-8 | CUFF.6995 | 918 | BGIBMGA004464 | nscaf2795: 2153376-2155029 | JN977526 | |  |
| Bm30K-9 | CUFF.6996 | 871 | BGIBMGA004465 | nscaf2795: 2164636-2165506 | JN977527 | |  |
| Bm30K-10 | CUFF.23775 | 916 | BGIBMGA004394 | nscaf2795: 1681447-1683203 | JN977528 | |  |
| Bm30K-11 | CUFF.10101 | 822 | BGIBMGA004457 | nscaf2795: 1615187-1616221 | JN977529 | |  |
| Bm30K-12 | CUFF.16801 | 1426 | BGIBMGA010876 | nscaf3005: 13956-15625 | JN977530 | |  |
| Bm30K-13 | CUFF.15264 | 1368 | BGIBMGA010168 | nscaf2986: 1478712-1480299 | JN977531 | |  |
| Bm30K-14 | CUFF.12499 | 1509 | BGIBMGA008164 | nscaf2891: 7548-9294 | JN977532 | |  |
| Bm30K-15 | CUFF.6800 | 1156 | BGIBMGA004454 | nscaf2795: 1559743-1560898 | JN977533 | |  |
| Bm30K-16 | CUFF.16776 | 967 | BGIBMGA010877 | nscaf3005: 17433-18399 | JN977534 | |  |
| **Bm30K-17^1^** | CUFF.23777 | **801** | **BGIBMGA004455** | **nscaf2795: 1590363-1591157** | **JN977535** | |  |
| Bm30K-18 | CUFF.15601 | 1330 | BGIBMGA004396 | nscaf2986: 1481449-1482778 | JN977536 | |  |
| Bm30K-19 | CUFF.12488 | 1472 | BGIBMGA008165 | nscaf2891: 11920-13391 | JN977537 | |  |
| **Bm30K-20^1^** | **CUFF.6841** | **836** | **BGIBMGA004387** | **nscaf2795: 2247170-2248005** | **JN977538** | |  |
| Bm30K-22 | CUFF.14791 | 1049 | BGIBMGA009573 | nscaf2962: 998075-999123 | JN977540 | |  |
| Bm30K-23 | CUFF.14792 | 1362 | BGIBMGA009621 | nscaf2962: 1000799-1002794 | JN977541 | |  |
| Bm30K-24 | CUFF.23783 | 1303 | BGIBMGA004398 | nscaf2795: 1637727-1640023 | JN977542 | |  |
| **Bm30K-25^1^** | CUFF. 23789 | **888** | **BGIBMGA004403** | **nscaf2795: 1582954-1583841** | **JN977543** | |  |
| **Bm30K-26^1^** | **CUFF.6843** | **1117** | **BGIBMGA004476** | **nscaf2795: 2342409-2343525** | **JN977544** | |  |
| Bm30K-28^2^ | CUFF.14731 | 414 | **——** | scaffold9081: 9-422 | JN977546 | |  |
| **Bm30K-29^1,2^** | **CUFF.22854** | **555** | **——** | **scaffold33722: 19-573** | **JN977547** | |  |

^1^New 30kDa protein family genes that were detected in our silkworm transcriptome.

^2^New 30kDa protein family genes found in the transcriptome that were not represented in the SilkDB; only the partial CDSs of these two genes were identified.

**Table S5-3 Dna J family of protein coding genes including the new potential genes (in bold) detected in our silkworm transcriptome.**

| **Protein name** | **mRNA ID** | **Length (bp)** | **SilkDB ID^b^** | **Position on**  **the genome** | **Accession number** |
| --- | --- | --- | --- | --- | --- |
| DnaJ1 | CUFF.9457 | 1806 | BGIBMGA006196 | nscaf2847:7317268-7319103 | JN872876 |
| DnaJ2 | CUFF.19321 | 2389 | BGIBMGA012541 | nscaf3048:558729-566236 | JN872877 |
| **DnaJ3^1^** | CUFF.4633 | **1202** | **BGIBMGA003153** | **nscaf2589:4200878-4202942** | **JN872878** |
| DnaJ4 | CUFF.10505 | 2380 | BGIBMGA006824 | nscaf2859:966713-975168 | JN872879 |
| **DnaJ5^1^** | CUFF.20841 | **1885** | **BGIBMGA013536** | **nscaf3075:951433-953711** | **JN872880** |
| DnaJ6 | CUFF.23327 | 1970 | BGIBMGA014455 | scaffold693:7605-11479 | JN872881 |
| DnaJ7 | CUFF.11803 | 1597 | BGIBMGA007808 | nscaf2888:1630480-1638035 | JN872882 |
| **DnaJ8^1^** | CUFF.4627 | **1252** | **BGIBMGA011314** | **nscaf3026:1160888-1162139** | **JN872883** |
| DnaJ9 | CUFF.9447 | 2596 | BGIBMGA006193 | nscaf2847:7256102-7264812 | JN872884 |
| DnaJ10 | CUFF.19337 | 1397 | BGIBMGA012569 | nscaf3048:1037184-1040328 | JN872885 |
| DnaJ11 | CUFF.19729 | 979 | —— | nscaf3058:2704812-2707332 | JN872886 |
| DnaJ12 | CUFF.16249 | 1460 | BGIBMGA010387 | nscaf2993: 6555160-6557408 | JN872887 |
| DnaJ13 | CUFF.13131 | 1025 | —— | nscaf2902: 8840618-8844805 | JN872888 |
| DnaJ14 | CUFF.14283 | 2311 | BGIBMGA009209 | nscaf2943: 2929098-2935664 | JN872889 |
| DnaJ15 | CUFF.13014 | 1100 | BGIBMGA008505 | nscaf2902:6382774-6384474 | JN872890 |
| DnaJ16 | CUFF.17873 | 2135 | BGIBMGA011435 | nscaf3027:4483222-4487459 | JN872891 |
| DnaJ17 | CUFF.760 | 2010 | BGIBMGA000581 | nscaf1690:1435582-1447224 | JN872892 |
| **DnaJ18^1^** | CUFF.711 | **1365** | **BGIBMGA000608** | **nscaf1690:208635-212919** | **JN872893** |
| DnaJ19 | CUFF.15356 | 2462 | BGIBMGA009967 | nscaf2980: 256364-271662 | JN872894 |
| DnaJ20 | CUFF.3328 | 1732 | BGIBMGA002188 | nscaf2216: 1884025-1891031 | JN872895 |
| DnaJ21 | CUFF.12433 | 899 | BGIBMGA008111 | nscaf2890: 916926-921870 | JN872896 |
| DnaJ22 | CUFF.19527 | 2848 | BGIBMGA012686 | nscaf3055: 56247-69007 | JN872897 |
| DnaJ23 | CUFF.19527 | 2090 | BGIBMGA012627 | nscaf3052: 1232560-1235439 | JN872898 |
| DnaJ24 | CUFF.21677 | 1343 | BGIBMGA014120 | nscaf463: 12836-15694 | JN872899 |
| DnaJ25 | CUFF.4271 | 2668 | BGIBMGA002821 | nscaf2575: 1417344-1436707 | JN872900 |
| **DnaJ26^1,3^** | CUFF.10145 | **786** | **BGIBMGA006645** | **nscaf2855: 2390949-2394008** | **JN872901** |
| **DnaJ27^1,2,3^** | CUFF.8080 | **378** | **BGIBMGA005229** | **nscaf2826: 240197-241311** | **JN872902** |

^1^New Dna J family genes that were detected in our silkworm transcriptome.

^2^New Dna J family genes found in the transcriptome that were not represented in the SilkDB.

^3^Only the partial CDSs of these genes were identified.

**Table S5-4** Transposase, Cuticle, Frizzled, Methuselah and APN family of protein coding genes detected in our silkworm transcriptome.

| **Protein name** | **mRNA ID** | **Length (bp)** | **SilkDB ID^b^** | **Position on**  **the genome** |
| --- | --- | --- | --- | --- |
| Transposase-1 | CUFF.17603 | 2538 | BGIBMGA011195 | nscaf3026: 5051837-5059246 |
| Transposase-2 | CUFF.1501 | 2612 | BGIBMGA009412 | nscaf1898: 4688556-4705686 |
| Cuticle-2 | CUFF.4472 | 730 | BGIBMGA003065 | nscaf2589: 1677516-1678708 |
| Cuticle-6 | CUFF.19479 | 453 | BGIBMGA012656 | nscaf3053: 179583-181548 |
| Frizzled-1 | CUFF.9487 | 2928 | BGIBMGA005935 | nscaf2847: 8021871-8025370 |
| Frizzled-4 | CUFF.11480 | 2584 | BGIBMGA007492 | nscaf2886: 1159192-1166865 |
| Methuselah-1 | CUFF.4165- CUFF.4167 | 2483 | BGIBMGA002851 | nscaf2575: 6743-10161 |
| Methuselah-2 | CUFF.7781; CUFF.7784 | 4772 | BGIBMGA004952 | nscaf2822: 198910-223119 |
| APN2 | CUFF.12349 | 2948 | BGIBMGA008062 | nscaf2889: 948509-960587 |
