# Supplementary material for: Transcriptome Analysis of the Silkworm (Bombyx mori) by High-Throughput RNA Sequencing
Source: PLoS One. 2012 Aug 23;7(8):e43713. doi: 10.1371/journal.pone.0043713 (PMC3426547; doi:10.1371/journal.pone.0043713)
Supplement: Table S6 — Primers used for new gene, alternative splicing and new exon validation. (DOC) [file pone.0043713.s010.doc]

**Supplementary Table S6** Primers used in this study.

|  | **Gene** | **Amplification (bp)** | **Primers** | **Sequences (5'→3')** |
| --- | --- | --- | --- | --- |
| RT-PCR validation | *Bm-Yellow-12* | 675 | F | CATAAAACTCTCGGTAACTTGATAGAG |
|  |  |  | R | CGGAAAGTGTTTTACCTCTTGGTTTAGAG |
|  | *Bm-Yellow-fa* | 721 | F | CCACGTTAAATTACGTAGATCTAACGAC |
|  |  |  | R | CCGACTTCGGCGTAAAACATTACGTTG |
|  | *Bm30K-17* | 750 | F | GTCACTCCAAAAATGAAGCCCG |
|  |  |  | R | ATGTTCGGGACTTCCCACGAC |
|  | *Bm30K-26* | 980 | F | GCAGTCAAGATGGCGTTCAAGC |
|  |  |  | R | GAAGGCAAGAATTTCAACAAAAATA |
|  | *DnaJ18* | 476 | F | CTGCAGCATTAGCATTTGACTCCTTCTC |
|  |  |  | R | GTATTTCCATGAATCGTTCTTGGGCAG |
|  | *DnaJ26* | 764 | F | GCTTCTTCTTCTTTCTCTTTCAGCTG |
|  |  |  | R | CATATTTTGTATTGTGAGAGCCAG |
|  | *Transposase-1* | 234 | F | ACTGCTGATGAACCTGCTCGAG |
|  |  |  | R | ACATATATCGGTGACAGTCCG |
|  | *Transposase-2* | 434 | F | AATTGCTGTTGCGCTTAATCTCGCT |
|  |  |  | R | ATATCGGTATGACTATACAAATGC |
|  | *Cuticle-2* | 294 | F | CAAGCGCACTGTGGACTACGC |
|  |  |  | R | GCGTATGTGCGACCATACAAAG |
|  | *Cuticle-6* | 299 | F | ACCGGTCCCAATGTGCATTCG |
|  |  |  | R | GTATATGAACTGCCGGATAAGG |
|  | *Frizzled-1* | 282 | F | GAAAGTAGATGGTGATGTACTGTCAG |
|  |  |  | R | ACTTGTTCATAGAACAAACATGCTATG |
|  | *Frizzled-4* | 296 | F | GTAGATGCCGATGAACTTACAGGTAC |
|  |  |  | R | CTCCACGCGTATTCATAAACCCAAGTAG |
|  | *Methuselah-1* | 389 | F | TCAAGTCTACATCCGAGAGGGCAG |
|  |  |  | R | TCAGCAGACCGTCTCCATGGGGAC |
|  | *Methuselah-2* | 805 | F | GATCCTGATGAGTATTGTCTCGGTG |
|  |  |  | R | CCATGATTTTCCAGATAATATGCG |
|  | *APN2* | 748 | F | GAAAGTAGATGGTGATGTACTGTCAG |
|  |  |  | R | GTCCCATCATGTTTCATGACAGTTC |
| Splice site vlidation | *CUFF.6110* | 330/343 | F1 | CGTTTCCTGTTTGACTATTATTAAAAAC |
|  |  |  | F2 | GCGTGTGCAGAGTATAGAATAATC |
|  |  |  | R | CTTGAGGTAGCTTGTCCTTGACGAC |
|  | *CUFF.12626* | 430/564 | F | GCGACTGGCAATGAAGTAACCAAC |
|  |  |  | R | GGAGGTCAATAAAGTTAGCTTCTC |
|  | *CUFF.13107* | 347/578/828 | F | CTGTACCCATCTCGCTACGGACTCTAC |
|  |  |  | R | GAGTAGCAGCAGTCTTTGAGATCTTG |
|  | *CUFF.5585* | 273/382 | F | GGAGTCATTCTTCTACACATTGCAAG |
|  |  |  | R | CAAAAAGACATGTGATTCTGTAGCAC |
|  | *Cuff.6350* | 608/417 | F1 | CGAATGCCCA ATAAGGAGTC GC |
|  |  |  | F2 | CAAATTCA CTCTTGAGTG AC |
|  |  |  | R | GATCATG TGGCGGCCTG CCCTC |
|  | *Cuff.10775* | 1080/1016 | F | CTATACCGT TTCCGCTCGT C |
|  |  |  | R1 | GTGGAGGTC TCTCAGGTGG ATG |
|  |  |  | R2 | CTTAGTCGAATTTCGTCGAGATGTG |
|  | *BGIBMGA007023* | 1032 | F | GCTGCTGGCAGAGGCTTTACAGAAC |
|  |  |  | R | CTGTAAGTATGACGGTAGACGTCCTTG |
|  | *BGIBMGA001040* | 320 | F | CGATTACGGGCCTGTCGCGCGCTCTC |
|  |  |  | R | GGATGCTAGAGCCGACAGCGAGTTC |
|  | *BGIBMGA010106* |  | F | CTGAAACA CAATATTAAGTTGAG |
|  |  |  | R | GGACTTTGTGATCTGGATTTAGG |
|  | *BGIBMGA010190* |  | F | CCATTATTT GAAGCTGATG TTC |
|  |  |  | R | GAGAGAACACGCACAGCCAACAG |
| Q-RT-PCR | *Frizzled1* | 177 | F | GAAAGTAGATGGTGATGTACTGTCAG |
|  |  |  | R | GTCCCATCATGTTTCATGACAGTTC |
|  | *Methuselah-1* | 177 | F | TCAAGTCTACATCCGAGAGGGCAG |
|  |  |  | R | CCACCACCAGGAAGATGAGCAC |
|  | *Yellow-12* | 187 | F | GTTATCATACAGCACTTAATGATGATTTC |
|  |  |  | R | GCTATAAGCATGTCTGTAAGTAGATC |
|  | *apn2* | 196 | F | CTAACGATCAGCTAGAAATGCTCAG |
|  |  |  | R | CTTATAGCAGCAATATTGTTCTTCAC |
|  | *Cuticle-2* | 294 | F | CAAGCGCACTGTGGACTACGC |
|  |  |  | R | GCGTATGTGCGACCATACAAAG |
|  | *Bm30K-26* | 205 | F | CTGTATGCGTTATGATCGACC |
|  |  |  | R | GGAAACACGATCTTGGTAGTTG |
|  | *Transposase-1* | 234 | F | ACTGCTGATGAACCTGCTCGAG |
|  |  |  | R | ACATATATCGGTGACAGTCCG |
